# Higher tumor mutational burden and PD-L1 expression correlate with shorter survival in hematologic malignancies

**DOI:** 10.1177/17588359241273053

**Published:** 2024-08-28

**Authors:** Ah-Reum Jeong, Aaron H. Trando, Sean D. Thomas, Paul Riviere, Patrick J. Sakowski, Ethan S. Sokol, Aaron M. Goodman, Razelle Kurzrock

**Affiliations:** Division of Blood and Marrow Transplantation, Department of Medicine, University of California San Diego, 3855 Health Sciences Drive, La Jolla, CA 92093-0658, USA; Division of Blood and Marrow Transplantation, Department of Medicine, University of California San Diego, La Jolla, CA, USA; University of California San Diego School of Medicine, La Jolla, CA, USA; Department of Radiation Medicine and Applied Sciences, University of California San Diego, La Jolla, CA, USA; Division of Blood and Marrow Transplantation, Department of Medicine, University of California San Diego, La Jolla, CA, USA; Foundation Medicine, Cambridge, MA, USA; University of California San Diego School of Medicine, La Jolla, CA, USA; Department of Medicine, Medical College of Wisconsin, 8800 West Doyne Avenue, Milwaukee, WI 53226, USA

**Keywords:** checkpoint inhibitors, hematologic malignancies, immunotherapy, PD-1 inhibitors, PD-L1 inhibitors, tumor mutational burden

## Abstract

**Background:**

The prognostic implications of tumor mutational burden (TMB) and programmed death ligand 1 (PD-L1) expression are poorly studied in hematologic malignancies.

**Objectives:**

This study aimed to better understand the characteristics and prognostic value of TMB and PD-1/PD-L1 in hematologic malignancies.

**Design:**

This real-world study was conducted among patients with hematologic malignancies who had next-generation sequencing (NGS) (Foundation Medicine) at the University of California San Diego Moores Cancer Center (2014–2018).

**Methods:**

TMB was measured by NGS. PD-L1 expression (tumor proportion score, TPS) was measured by immunohistochemistry (classified as high (⩾50%), low (1–49%), and negative (<1%)). Data was curated from the electronic medical records.

**Results:**

In 388 evaluable patients, the most common diagnoses were B-cell non-Hodgkin lymphoma (NHL) (35%) and Philadelphia chromosome-negative myeloproliferative disorders (16%). Median TMB was 1.6 mutations/Mb (range, 0–46.83). Forty-eight patients (12%) had TMB ⩾10 mutations/Mb, 90% of which were B-cell or T-cell NHL. In 85 samples with available PD-L1 scores, 11 were high; 26, low; and 48, no tumor cell expression. PD-L1 TPS positive (⩾1%) was most common in T-cell NHL (7/9 (77%) cases) followed by B-cell NHL (21/51 (41%) cases). TMB ⩾4 mutations/Mb and PD-L1 score ⩾1% were significantly associated with shorter overall survival (OS) from diagnosis, with hazard ratio (HR) = 1.46 (*p* = 0.02, 95% confidence interval (CI) 1.05–2.03) and HR = 2.11 (*p* = 0.04, 95% CI 1.04–4.30), respectively; the relationship was more pronounced when PD-L1 ⩾50% versus <50% was used (HR = 2.80, *p* = 0.02, 95% CI 1.19–6.59). Higher TMB and higher PD-L1 positivity correlation were significant but weak (Pearson correlation coefficient *R*^2^ = 0.04, *p* = 0.04).

**Conclusion:**

TMB ⩾4 mutations/Mb and positive PD-L1 TPS are poor prognostic factors, correlating with shorter OS across hematologic malignancies.

**Trial registration:**

ClinicalTrials.gov NCT02478931.

## Introduction

Tumor mutational burden (TMB) and programmed death 1 (PD-1) and its ligand (PD-L1) expression are two well-established biomarkers that are predictive of response to immune checkpoint inhibitor (ICI) therapy.^1–6^ These biomarkers have well-defined prognostic and predictive implications in solid tumors, but their prognostic role in hematologic malignancies is considerably less studied.

The increased TMB of a cancer cell’s DNA is suspected to increase neoantigen presentation via the major histocompatibility complex (MHC), which may allow for better recognition by the immune system upon activation by ICIs. Meanwhile, the PD-1/PD-L1 pathway is one of the inhibitory receptors of the immune system that is exploited by cancer cells to evade immune surveillance; blocking this pathway leads to a cytotoxic antitumor response.^7^ TMB and PD-1/PD-L1 expression are routinely assessed and used to guide therapeutic decisions in solid malignancies. TMB ⩾10 mutations/Mb is a tissue-agnostic biomarker that predicts response to pembrolizumab, as demonstrated in the pivotal clinical trial KEYNOTE-158,^8^ resulting in its approval by the Food and Drug Administration for this purpose.^9^ TMB ⩾16 mutations/Mb appears to be predictive of response in patients treated with atezolizumab.^10^

The prognostic role of TMB and PD-1/PD-L1 expression is less clear. We previously demonstrated that low TMB of <5 mutations/Mb and high TMB of ⩾20 mutations/Mb portends better prognosis in ICI naïve patients while intermediate TMB, defined as >5 mutations/Mb and <20 mutations/Mb, correlates with worse prognosis.^11^ Studies demonstrate that the immune signature in cancers with low and high TMB can differ depending on the type of cancer, which could explain some inconsistencies in the association between TMB and ICI outcomes.^12^ Furthermore, increased PD-L1 expression is correlated with worse prognosis in multiple solid cancer types.^13^

A deeper understanding of the predictive role of TMB and PD-1/PD-L1 in treatment with ICI has led to breakthrough treatment options for solid tumors, but their efficacy in hematologic malignancies has been mixed. Currently, checkpoint inhibitors are only FDA-approved for two hematologic indications: relapsed/refractory classical Hodgkin lymphoma (R/R cHL) and relapsed/refractory primary mediastinal B-cell lymphoma (PMBCL).^14^ In both of these conditions, checkpoint inhibitors have been demonstrated to be at least as effective, if not more, than in their solid organ counterparts. In R/R cHL clinical trials, CHECKPOINT 205^15^ demonstrated an objective response rate (ORR) of 69% with single-agent nivolumab while KEYNOTE-087^16^ yielded an ORR of 71.9% with pembrolizumab. The ORR observed in KEYNOTE-013 and KEYNOTE-170,^17^ two clinical trials evaluating pembrolizumab monotherapy in R/R PMBCL, was 48% and 45%, respectively. The remarkable response in cHL and PMBCL is attributed to their unique genetic properties that include copy number alterations of 9p24.1, on which the PD-L1 gene resides.^17–20^ In contrast, lower response rates to single-agent checkpoint inhibitor therapy have been observed among most non-Hodgkin lymphomas (NHLs), multiple myeloma (MM), and leukemias, with ORRs of 3–33%.^19,21–23^

The TMB and PD-1/PD-L1 characteristics in hematologic malignancies remain poorly defined, let alone their prognostic and predictive roles. These have been best described in cHL in which 9p24.1 amplification in cHL is associated with higher expression of PD-L1 and worse progression-free survival (PFS).^24^ Similar to solid malignancies, higher PD-L1 expression in cHL has been associated with improved prognosis when treated with nivolumab.^18^ Therefore, it is important to understand the characteristics of TMB and PD-1/PD-L1 in hematologic malignancies, which we explore in this report.

## Methods

This study was conducted in accordance with the University of California San Diego Internal Review Board-approved PREDICT study (NCT02478931) and any investigational interventions for which patients consented. All patients had hematologic malignancies treated at the University of California San Diego Moores Cancer Center and had next-generation sequencing (NGS) on the FoundationOne^®^ Heme panel performed between 2014 and 2018. A review of the electronic medical records was performed. This was a real-world retrospective study and included all patients with available clinical-grade TMB data who participated in the PREDICT study. The reporting of this study conforms to the Strengthening the Reporting of Observational Studies in Epidemiology statement^25^ (Supplemental Table 1).

Tumor tissue profiling by massively parallel NGS of 406 genes and select introns of 31 genes involved in rearrangements as well as RNA sequencing (cDNA) of 265 commonly rearranged genes fusions were performed using the FoundationOne Heme assay (Foundation Medicine, Cambridge, MA, USA) as described previously.^26,27^ TMB was determined on 1.2 megabases of DNA sequence, excluding germline and driver alterations from the calculation.^28^ Microsatellite instability (MSI) was determined by examining homopolymer repeat regions.^29^

PD-L1 scores of the tumor were classified as high (⩾50%), low (1–49%), and negative (<1%). PD-L1 tumor proportion score (TPS) was determined by immunohistochemistry (IHC) performed at Foundation Medicine. The assay utilized Dako (22C3) or Ventana (SP142) anti-PD-L1 antibodies and was performed according to manufacturer instructions on Formalin-fixed, paraffin-embedded (FFPE) tissue of the tumor.

Descriptive statistics were utilized to summarize patient characteristics. PFS and overall survival (OS) were visualized using Kaplan–Meier and quantified with Cox proportional hazards regression reporting two-sided *p*-values. Correlation of the TMB and PD-L1 score was assessed by Pearson correlation coefficient, and the difference between the medians of TMB by disease group was calculated using one-way analysis of variance (ANOVA). A confidence interval (CI) of 95% was used for all analyses.

## Results

A total of 480 patients were identified. After applying the exclusion criteria, 388 patients were included in final analysis (Supplemental Figure 1). The median age at diagnosis was 61 years and 55.8% were men. The most common diagnosis was B-cell NHL (35%) followed by Philadelphia chromosome-negative myeloproliferative neoplasm (MPN) disorders (16%) and myelodysplastic syndrome (MDS) (12%). None of the 302 patients who were tested for MSI status were MSI-High. TMB and PD-L1 scores are shown in Table 1.

**Table 1.** TMB and tumor PD-L1 scores of all patients.

Disease type	TMB		PD-L1 TPS^a^			TMB ⩾4 mut/Mb and/or PD-L1 positive
	⩾4 mut/Mb	<4 mut/Mb	High (⩾50%)	Low (1–49%)	Negative (<1%)	
All diagnosis (*n* = 388)	120/388 (31%)	269/388 (69%)	11/85 (13%)	26/85 (31%)	48/85 (56%)	131/388 (34%)
B-ALL (30)	6/30 (20%)	24/30 (80%)	0	2/7 (29%)	5/7 (71%)	7/30 (23%)
T-ALL (6)	1/6 (17%)	5/6 (83%)	0	0	0	1/6 (17%)
AML (38)	8/38 (21%)	30/38 (79%)	0	2/4 (50%)	2/4 (50%)	8/38 (21%)
HL (2)	0	2/2 (100%)	1/2 (50%)	1/2 (50%)	0	2/2 (100%)
B-NHL (136)	77/136 (57%)	59/136 (43%)	6/51 (12%)	15/51 (29%)	30/51 (59%)	81/136 (60%)
T-NHL (26)	10/26 (38%)	16/26 (62%)	3/9 (33%)	4/9 (44%)	2/9 (22%)	12/26 (46%)
MM (19)	10/19 (53%)	9/19 (47%)	0	0	3/3 (100%)	10/19 (53%)
MDS (45)	3/45 (7%)	42/45 (93%)	0	0	2/2 (100%)	3/45 (7%)
CML (9)	0	9/9 (100%)	0	0	0	0
MPN, Ph neg (61)	2/61 (3%)	59/61 (97%)	0	1/2 (50%)	1/2 (50%)	3/61 (5%)
MDS/MPN (9)	0	9/9 (100%)	0	0	0	0
Other^b^ (7)	2/7 (29%)	5/7 (71%)	1/5 (20%)	1/5 (20%)	3/5 (60%)	4/7 (57%)

a Samples were either stained with Dako 22C3 PD-L1 antibody or Ventana SP142 PD-L1 antibody.

b Including acute leukemia of ambiguous lineage (*n* = 2), histiocytic malignancies (*n* = 2), post-transplant lymphoproliferative disorder (*n* = 3).

AML, acute myeloid leukemia; B-ALL, B-cell acute lymphoblastic leukemia; B-NHL, B-cell non-Hodgkin lymphoma; CLL, chronic lymphocytic leukemia; CML, chronic myeloid leukemia; MDS, myelodysplastic syndrome; MM, multiple myeloma; MPN, myeloproliferative neoplasm; N/A, not available; neg, negative; PD-L1, programmed death ligand 1; Ph, Philadelphia chromosome; SLL, small lymphocytic lymphoma; T-ALL, T-cell acute lymphoblastic leukemia; TMB, tumor mutational burden; T-NHL, T-cell non-Hodgkin lymphoma; TPS, tumor proportion score.

Seventeen patients received checkpoint inhibitor combination therapy as part of various clinical trials. The baseline characteristics, treatment, and outcomes are described in Table 2. One patient had PMBCL while none of the patients had cHL. The overall response rate, defined as patients who achieved partial response (PR) or better, was 24% (4 of 17 patients), with one patient with peripheral T-cell lymphoma (PTCL) achieving complete response (CR). The other three patients with PR had Richter’s transformation to diffuse large B-cell lymphoma (DLBCL), primary central nervous system lymphoma (PCNSL), and gray zone lymphoma (GZL). None of the patients with T-cell lymphoma experienced hyperprogression. The median PFS from the first dose of checkpoint therapy to the date of discontinuation due to toxicity, progression, or death was 78 days. The four patients who responded had a PFS of 951, 580, 567, and 193 days; one additional patient had a mixed response lasting 300 days. The TMB of the responders was 0.8, 4.8, 16.1, and 40.3 mutations/Mb; the patient with a TMB of 4.8 achieved a CR. The patient with a mixed response lasting 300 days had a TMB of 32.4. Five of the 12 other patients had a TMB >10 mutations/Mb. PD-L1 TPS was 1, 10, and 50 in the responders with available data. The small number of patients and diagnostic heterogeneity of the groups precluded statistical analysis.

**Table 2.** Characteristics and outcomes of patients who received immune checkpoint inhibitor therapy.

Pt No	Disease	TMB (mut/Mb)	PD-L1 TPS (%)	Treatment	Best response	PFS (days)^a^
1	DLBCL	25.0	50	Nivolumab + STAT3 inhibitor	Unknown	34
2	DLBCL	13.7	1	Nivolumab + STAT3 inhibitor	Progressive disease	90
3	Transformed DLBCL	11.3	60	Pembrolizumab + venetoclax	Progressive disease	48
4	Richter’s transformation DLBCL	0.8	10	Nivolumab + ibrutinib	Partial response	951
5	PMBCL	17.0	0	Pembrolizumab + BV	Stable disease	124
6	GZL	16.1	50	Pembrolizumab + BV + SBRT	Partial response	580
7	PCNSL	40.4	N/A	Nivolumab + temozolomide	Partial response	567
8	ALK (−) PTCL	11.3	10	Pembrolizumab	Unknown	9
9	PTCL	2.4	N/A	Nivolumab + bendamustine	Mixed response	61
10	PTCL	4.8	1	Nivolumab + trametinib + anakinra	Complete response	193
11	Cutaneous γδ T-cell lymphoma	7.3	N/A	Nivolumab + BV	Mixed response	70
12	NK T-cell lymphoma	4.8	25	Nivolumab	Progressive disease	174
13	Mycosis fungoides	32.3	0	Pembrolizumab + RT	Mixed response	300
14	HIV-related plasmablastic lymphoma	1.0	60	Pembrolizumab + BV	Progressive disease	68
15	Multiple myeloma	4.8	N/A	Pembrolizumab + pomalidomide + dexamethasone	Stable disease	78
16	Multiple myeloma	0.0	N/A	Pembrolizumab + pomalidomide + dexamethasone	Unknown	14
17	AML	6.5	N/A	Pembrolizumab	Progressive disease	13

a PFS from first dose of checkpoint treatment to progression or death.

ALK, anaplastic lymphoma kinase; AML, acute myeloid leukemia; BV, brentuximab vedotin; DLBCL, diffuse large B-cell lymphoma; GZL, gray zone lymphoma; HIV, human immunodeficiency virus; Mb, megabase; N/A, not available; NK, natural killer; PCNSL, primary central nervous system lymphoma; PD-L1, programmed death 1 ligand; PFS, progression-free survival; PMBCL, primary mediastinal B-cell lymphoma; PTCL, peripheral T-cell lymphoma; SBRT, stereotactic body radiation therapy; STAT3, signal transducer and activator of transcription 3; TMB, tumor mutational burden; TPS, tumor proportion score.

**Figure 1.**
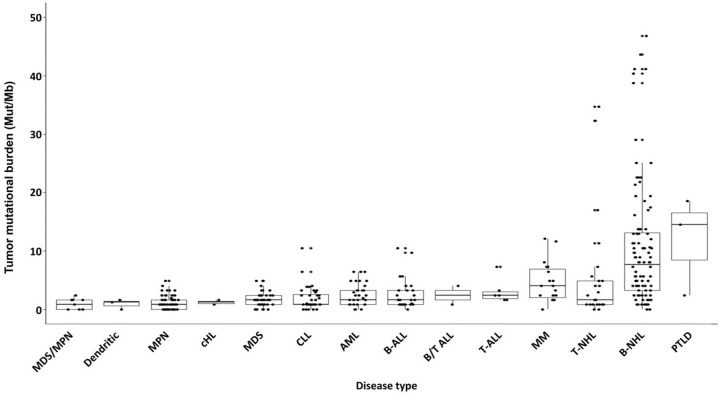
Box plot of TMB by disease type. The middle line represents the median and box represents interquartile range. Each dot represents one tumor sample. B-NHL excludes CLL. The median values were significantly different by one-way ANOVA (*F* = 11.7, *p* < 0.001). AML, acute myeloid leukemia; B-ALL, B-cell acute lymphoblastic leukemia; B/T-ALL, acute leukemia of ambiguous lineage; B-NHL, B-cell non-Hodgkin lymphoma; cHL, classic Hodgkin lymphoma; CLL, chronic lymphocytic leukemia; MDS, myelodysplastic syndrome; MM, multiple myeloma; MPN, myeloproliferative neoplasm; PD-L1, programmed death 1 ligand; PTLD, post-transplant lymphoproliferative disorder; T-ALL, T-cell acute lymphoblastic leukemia; TMB, tumor mutational burden; T-NHL, T-cell non-Hodgkin lymphoma; TPS, tumor proportion score.

TMB differed by hematologic disease type: The median TMB of the entire cohort was 1.6 mutations/Mb. The median TMB of B-cell NHL was 4.0 mutations/Mb, and when chronic lymphocytic leukemia (CLL), which had a median TMB of 0.8 mutations/Mb, was excluded, the median was 7.7 mutations/Mb. The median TMB in T-cell NHL was 1.6 mutations/Mb and that of MM was 4.0 mutations/Mb. There was a significant difference in median TMB by disease type (one-way ANOVA, *F* = 11.7, *p* < 0.001) (Figure 1). Post-transplant lymphoproliferative disorder had the highest median TMB (14.5 mutations/Mb) while MDS/MPN overlap syndrome had the lowest median TMB (0.8 mutations/Mb).

When examining lymphoid and myeloid malignancies separately (Supplemental Figure 2), TMB ⩾4 mutations/Mb versus <4 mutations/Mb was prognostic for significantly worse survival in lymphoid malignancies (hazard ratio (HR) = 1.55, 95% CI 1.02–2.36, *p* = 0.04); in myeloid malignancies, the median OS was 23.4 versus 198.8 months for TMB ⩾4 mutations/Mb versus <4 mutations/Mb (HR = 1.90, 95% CI 0.90–4.00, *p* = 0.087). The latter value may not have reached statistical significance despite the much different median OS because of the small number of patients (*n* = 13) in the TMB ⩾4 mutations/Mb group.

**Figure 2.**
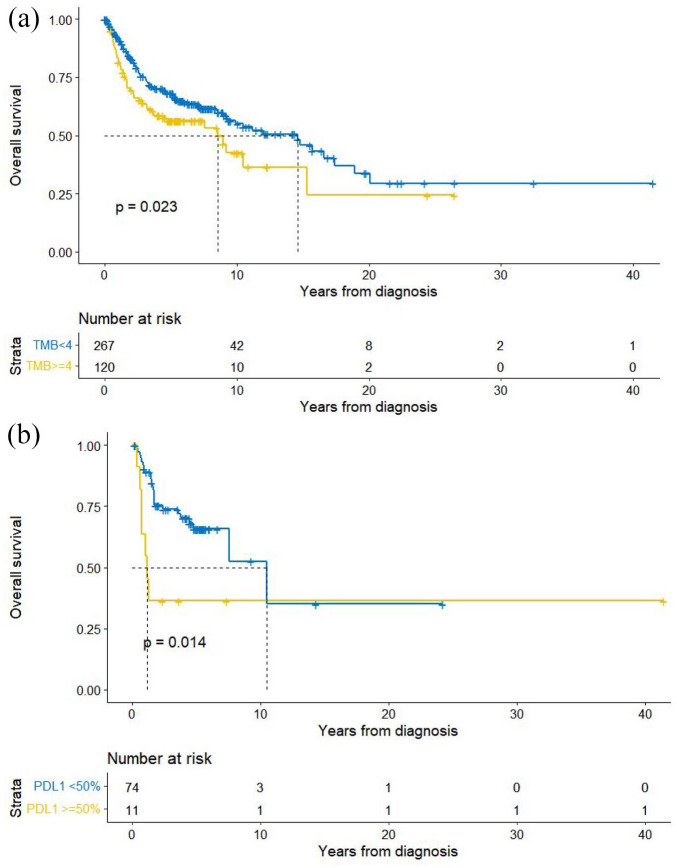
Kaplan–Meier survival curves based on TMB and PD-L1 TPS. (a) Kaplan–Meier curve of OS from date of diagnosis by TMB ⩾4 mutations/Mb versus <4 mutations/Mb (HR = 1.46, *p* = 0.02, 95% CI 1.05–2.03). Patients with higher TMB had significantly shorter survival. (b) Kaplan–Meier curve of OS from date of diagnosis by PD-L1 TPS score ⩾50% versus <50% (HR = 2.80, *p* = 0.02, 95% CI 1.19–6.59). Patients with PD-L1 TPS score ⩾50% had a significantly shorter survival. CI, confidence interval; HR, hazard ratio; OS, overall survival; PD-L1, programmed death ligand 1; TMB, tumor mutational burden; TPS, tumor proportion score.

Higher TMB (⩾4 mutations/Mb) correlated with shorter OS from diagnosis: Importantly, we tested the lower TMB cutoff of ⩾4 mutations/Mb, given a previous prognostic report in DLBCL,^30^ and discovered it had significantly shorter OS compared to <4 mutations/Mb (HR = 1.46, *p* = 0.02, 95% CI 1.05–2.03) (Figure 2(a)). Among all patients, 31% of patients had ⩾4 mutations/Mb and 69% had <4 mutations/Mb (Table 1).

There was no significant difference in OS from the time of diagnosis by a TMB cutoff of 10 mutations/Mb (⩾10 vs <10 mutations/Mb) (cutoff used by FDA for tumor-agnostic approval of pembrolizumab)^9^ (HR = 1.19, *p* = 0.45, 95% CI 0.75–1.91). Furthermore, while TMB ⩾16 mutations/Mb has been shown to predict better outcomes after ICI therapy,^10^ there was no significant difference in OS with a cutoff of 16 mutations/Mb (HR = 1.55, *p* = 0.2, 95% CI 0.84–2.87), possibly related to the low number of cases with TMB ⩾16 mutations/Mb (24 of 388, or 6.2%). We could not investigate the parabolic relationship between TMB and survival as described previously^11^ wherein intermediate TMB had worse prognosis in patients who never received immunotherapy as compared to low (⩽5 mutations/Mb) and very high TMB (⩾50 mutations/Mb), because none of the patients in this cohort had very high TMB.

High PD-L1 TPS correlated with shorter OS from diagnosis: PD-L1 TPS scores were available for 85 patients (Table 1) and were high (⩾50%) in 11 (13%), low (1–49%) in 26 (31%), and negative (<1%) in 48 (56%) cases. Positive PD-L1 TPS (⩾1%) was most common in T-cell NHL (7 of 9 (77%) cases) followed by 21 of 51 (41%) cases of B-cell NHL. Patients with positive PD-L1 TPS had significantly shorter OS (HR = 2.11, *p* = 0.04, 95% CI 1.04–4.30), which was even more pronounced when ⩾50% versus <50% was used (HR = 2.80, *p* = 0.02, 95% CI 1.19–6.59) (Figure 2(b)).

Correlation between TMB and PD-L1 expression: Similar to a prior study,^31^ the correlation between higher TMB and higher PD-L1 positivity was statistically significant, but weak (Pearson correlation coefficient *R*^2^ = 0.04, *p* = 0.04) (data not shown).

## Discussion

In this study, we describe the characteristics of TMB and PD-L1 TPS in various hematologic malignancies and explore their prognostic value.^1,2,4–6,32^ We noted that the TMB in hematologic malignancies tends to be low, especially among the myeloid malignancies. The median TMB was higher in B- and T-cell NHL. If the conventional cutoff of TMB ⩾10 mutations/Mb is used, as therapeutically relevant given the FDA approval for pembrolizumab,^9^ only 12% of all tumors were TMB-high, 90% of which were B-cell or T-cell NHL. Similar to TMB-high status, a positive PD-L1 status in our study was observed mostly in patients with lymphoma. Notably, two of two cHL were positive for PD-L1, which is predicted by the 9p24.1 copy number alteration characteristically seen in cHL.^33^

TMB in various lymphoid malignancies has been poorly described in the literature, but there are a few notable studies. Cho et al.^34^ analyzed single nucleotide variants (SNVs) and insertion/deletions (indels) of 405 genes in 300 patients with NHL. Mature B-cell neoplasms had an average number of SNVs/indels of 23.98 and mature T-cell neoplasms had an average of 17.21. PMBCL had the highest median number of SNVs/indels (32), followed by PCNSL (30), and DLBCL NOS (23)/anaplastic lymphoma kinase-negative Anaplastic Large Cell Lymphoma (ALCL) (23). The study also demonstrated that there were no differences in number of mutations according to initial diagnosis, time of relapse, or stage in most of the lymphomas. Ou et al.^35^ reported the TMB and PD-L1 expression in 48 patients with PCNSL; 66.7% of patients had positive PD-L1 expression and 41 of 42 patients had TMB ⩾5 mutations/Mb. Another study demonstrated that 23 of 34 patients with cHL had TMB ⩾5 mutations/Mb.^36^

Given the generally low TMB observed in hematologic malignancies, we explored the cutoff value of ⩾4 mutations/Mb used in a previous report by Chen et al.^30^ who analyzed TMB from a 69-gene panel NGS in 87 patients with DLBCL. The authors demonstrated that patients with tumor TMB ⩾4 mutations/Mb had poor survival compared to those with tumor TMB <4 mutations/Mb. With this lower cutoff, we also demonstrated that patients with higher TMB had shorter survival from diagnosis across hematologic malignancies. These results are also similar to those in solid tumors, in which TMB > 5 is associated with worse prognosis than TMB ⩽5 mutations/Mb.^11^

We also investigated the prognostic value of PD-L1 TPS for survival. In our entire cohort of samples, a positive PD-L1 TPS score was correlated with worse prognosis compared to a negative PD-L1 TPS score; the patients with PD-L1 ⩾50% had a particularly worse prognosis even when limited by the small number of patients in this group. It is plausible that this worse prognosis is due to the immune system shield that PD-L1 provides. There are several other studies that describe PD-L1 expression in lymphoma, which vary widely in their results. Tumor PD-L1 score is reported to be positive in 10–50% of DLBCL, 70–100% of PMBCL, 0–10% of CLL, and 7–80% of T-cell NHL.^37^ The prognostic value of PD-L1 score in NHL is also unclear, with some studies reporting worse OS and some suggesting no difference.^37^

A total of 17 patients who had positive biomarkers (either high TMB and/or positive PD-L1 expression) were treated with ICI-based therapy. Notably, all but one patient received combination therapy with other agents. Only 4 of these 17 patients achieved an objective response. The four patients who responded had PFS values ranging from 193 to 951 days. Three of the four patients had B-cell NHL and 1 had T-cell NHL. Importantly, the patient with GZL received brentuximab vedotin and a patient with PCNSL received temozolomide, which are active agents in their respective diseases. It appears that a select group of patients derive benefit from ICI combination therapy.

There are several limitations to this study. Since NGS is not routinely obtained for many hematologic malignancies, patients who were enrolled in this study and had NGS data available are likely to suffer from selection bias. NGS was also obtained at different time points of the disease, with some obtained at the time of diagnosis and others at the time of relapse, although our previous work suggests that TMB remains constant from diagnosis to relapse.^38^ Another limitation is that the PD-L1 IHC testing was performed using two different antibodies, Dako (22C3) and Ventana (SP142), which may lead to discrepancies in results. Our dataset is also limited by the relatively small sample size; as such, detailed assessments stratified by individual cancer types were not able to be conducted, though our results may suggest applicability across tumor types, especially since similar results (i.e. better prognosis with lower TMB) have been reported in solid tumors.^11^ Another limitation of the current work is that PD-L1 expression was examined in tumor cells; further studies should also interrogate the microenvironment. Finally, the ICI-treated patient number was small and heterogeneous, allowing for descriptive but not statistical analysis.

## Conclusion

In summary, this study describes detailed characteristics of TMB and PD-L1 expression for various hematologic malignancies. Acute leukemias and myeloid malignancies had low TMB while a subset of lymphomas had higher TMB and/or positive PD-L1 expression. TMB cutoff of ⩾4 mutations/Mb and positive PD-L1 TPS scores (especially scores ⩾50%) were each associated with significantly shorter survival. These data suggest that TMB and PD-L1 expression may be important biomarkers for the prognosis of hematologic malignancies in addition to their implications for solid tumors.^11,39,40^

## Supplemental Material

sj-docx-1-tam-10.1177_17588359241273053 – Supplemental material for Higher tumor mutational burden and PD-L1 expression correlate with shorter survival in hematologic malignancies Supplemental material, sj-docx-1-tam-10.1177_17588359241273053 for Higher tumor mutational burden and PD-L1 expression correlate with shorter survival in hematologic malignancies by Ah-Reum Jeong, Aaron H. Trando, Sean D. Thomas, Paul Riviere, Patrick J. Sakowski, Ethan S. Sokol, Aaron M. Goodman and Razelle Kurzrock in Therapeutic Advances in Medical Oncology

sj-docx-2-tam-10.1177_17588359241273053 – Supplemental material for Higher tumor mutational burden and PD-L1 expression correlate with shorter survival in hematologic malignancies Supplemental material, sj-docx-2-tam-10.1177_17588359241273053 for Higher tumor mutational burden and PD-L1 expression correlate with shorter survival in hematologic malignancies by Ah-Reum Jeong, Aaron H. Trando, Sean D. Thomas, Paul Riviere, Patrick J. Sakowski, Ethan S. Sokol, Aaron M. Goodman and Razelle Kurzrock in Therapeutic Advances in Medical Oncology

## Declarations

**Ethics approval and consent to participate:** The study was conducted in accordance with the Declaration of Helsinki and conducted in accordance with the University of California San Diego Internal Review Board-approved PREDICT study (NCT02478931) and any investigational interventions for which patients consented.

**Consent for publication:** Not applicable.

**Availability of data and materials:** The datasets used and/or analyzed during the current study are available from the corresponding author on reasonable request.
