# Supplementary material for: Higher tumor mutational burden and PD-L1 expression correlate with shorter survival in hematologic malignancies
Source: Ther Adv Med Oncol. 2024 Aug 28;16:17588359241273053. doi: 10.1177/17588359241273053 (PMC11363031; doi:10.1177/17588359241273053)
Supplement: sj-docx-1-tam-10.1177_17588359241273053 – Supplemental material for Higher tumor mutational burden and PD-L1 expression correlate with shorter survival in hematologic malignancies [file sj-docx-1-tam-10.1177_17588359241273053.docx]

Supplemental Figure 1. Flow diagram

**Abbreviations:** MGUS= monoclonal gammopathy of uncertain significance; NGS= next generation sequencing; WHO=World Health Organization.


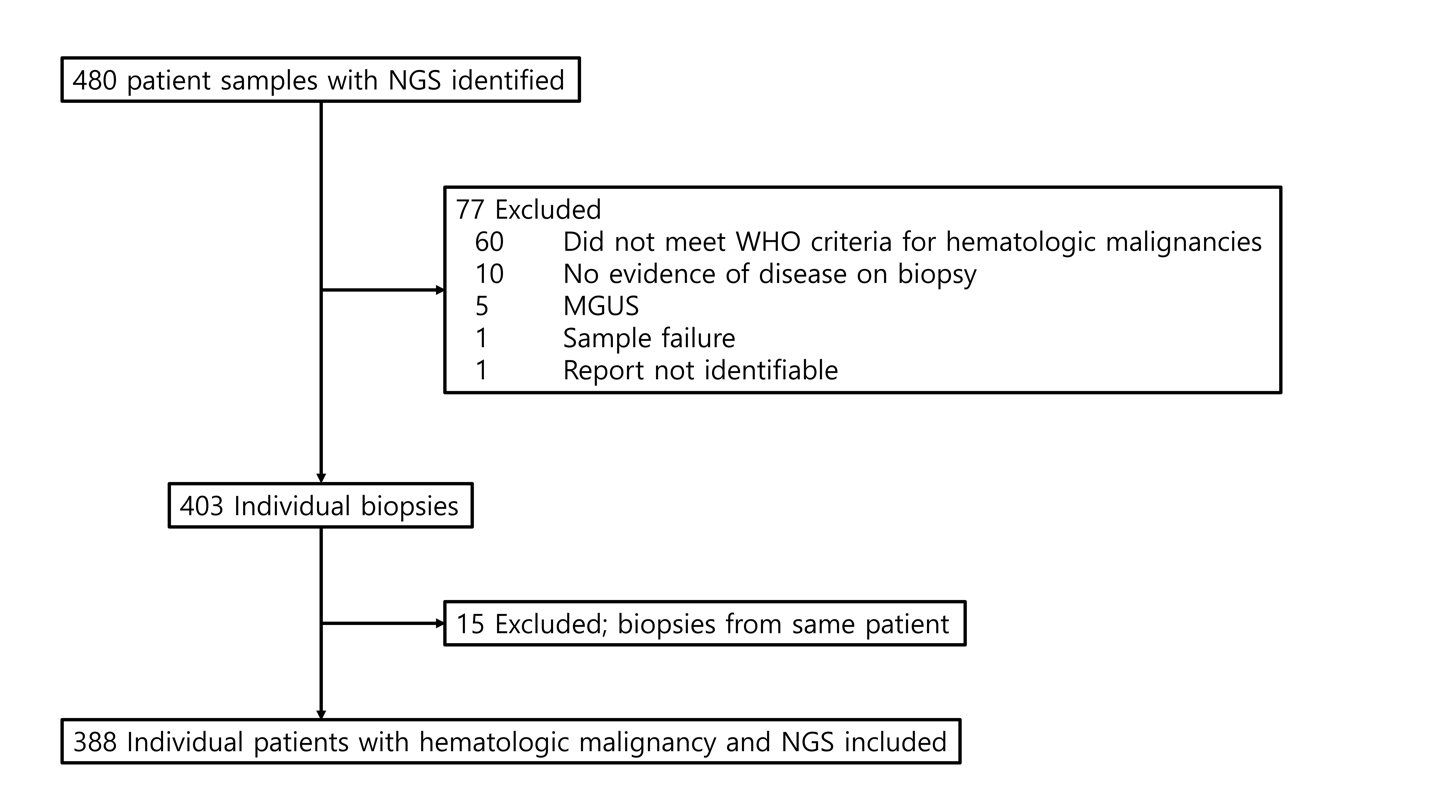


A.


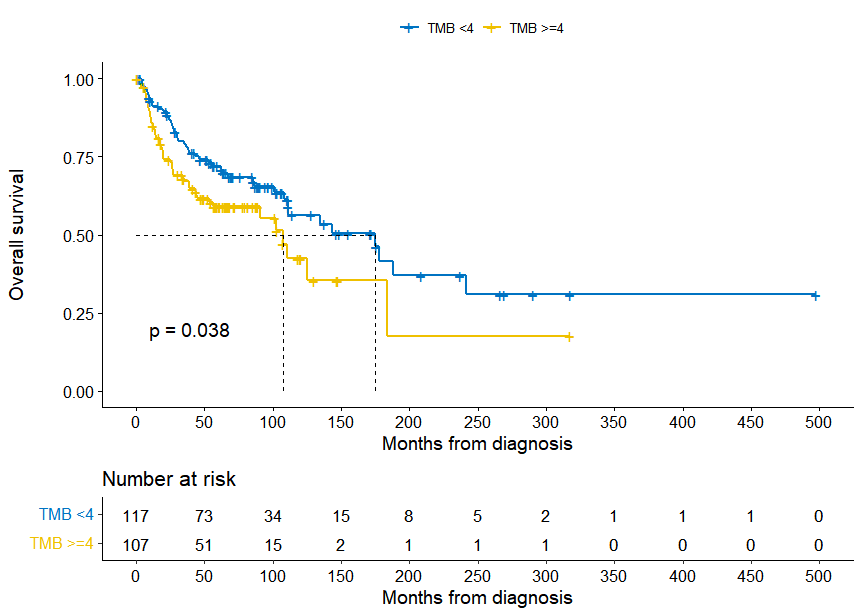


B.


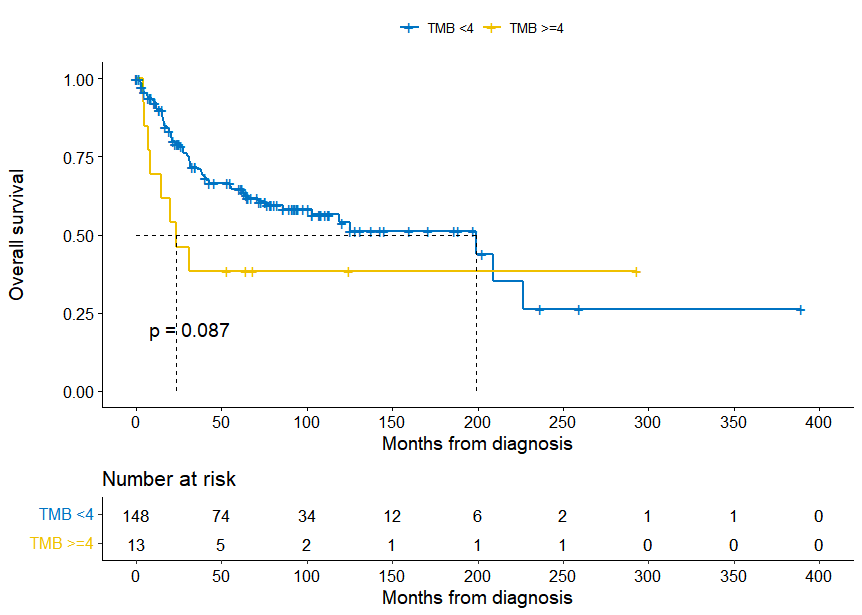


**Supplemental Figure 2**. **Kaplan Meier survival curves based on lymphoid and myeloid malignancies subtypes.** Panel A: Kaplan Meier curve of OS from date of diagnosis by TMB ≥4 mutations/Mb versus <4 mutations/Mb in all lymphoid malignancies (HR=1.55, p=0.04, 95% CI 1.02-2.36). Patients with higher TMB had a significantly shorter survival. Panel B: Kaplan Meier curve of OS from date of diagnosis by TMB ≥4 mutations/Mb versus <4 mutations/Mb in all myeloid malignancies (HR=1.90, p=0.087, 95% CI 0.90-4.00).
